# Comparison of antibiofilm activity of low-concentrated hypochlorites vs polyhexanide-containing antiseptic

**DOI:** 10.3389/fcimb.2023.1119188

**Published:** 2023-03-15

**Authors:** Justyna Paleczny, Adam Felix Junka, Paweł Krzyżek, Joanna Czajkowska, Axel Kramer, Hicham Benkhai, Ewa Żyfka-Zagrodzińska, Marzenna Bartoszewicz

**Affiliations:** ^1^ Department of Pharmaceutical Microbiology and Parasitology, Unique Application Models Laboratory, Wroclaw Medical University, Wroclaw, Poland; ^2^ Department of Microbiology, Wroclaw Medical University, Wroclaw, Poland; ^3^ Department of Biochemistry and Molecular Biology, Wroclaw University of Environmental and Life Sciences, Wroclaw, Poland; ^4^ Institute of Hygiene and Environmental Medicine, University Medicine Greifswald, Greifswald, Germany; ^5^ Faculty of Medicine, Lazarski University, Warsaw, Poland

**Keywords:** wound antisepsis, hypochlorite, polyhexanide, antibiofilm activity, *Pseudomonas aeruginosa*, *Staphylococcus aureus*, *Candida albicans*, *in vitro* biofilm models

## Abstract

Chronic wound infection is highly associated with morbidity and endangers the patient's life. Therefore, wound care products must have a potent antimicrobial and biofilm-eradicating effect. In this work, the antimicrobial/antibiofilm activity of two low-concentrated chlorine-based and releasing solutions was investigated on a total of 78 strains of methicillin-resistant *Staphylococcus aureus*, *Pseudomonas aeruginosa*, and *Candida albicans*, using the cohesive spectrum of *in vitro* settings, including microtiter plate models, biofilm-oriented antiseptic test, cellulose-based biofilm model, biofilm bioreactors and Bioflux model. The antiseptic containing polyhexamethylene biguanide was used in the character of usability control of performed tests. The results obtained by static biofilm models indicate that low-concentrated chlorine-based and releasing solutions display none to moderate antibiofilm activity, while data obtained by means of the Bioflux model, providing flow conditions, indicate the moderate antibiofilm activity of substances compared with the polyhexanide antiseptic. Considering *in vitro* data presented in this manuscript, the earlier reported favorable clinical results of low-concentrated hypochlorites should be considered rather an effect of their rinsing activity combined with low cytotoxicity but not the antimicrobial effect per se. For the treatment of heavily biofilm-infected wounds, polyhexanide should be considered the agent of choice because of its higher efficacy against pathogenic biofilms.

## 1 Introduction

The increasing volume of data indicates that multicellular communities of microorganisms, embedded within a protective extracellular matrix, referred to as the biofilms, are one of the major factors of chronic wounds’ persistence. Such a constatation forced the re-formulation of preventive and therapeutic recommendations for chronic wound treatment (Probst et al., 2022). The present data indicate that the application of locally-administered antiseptic agents should be considered one of the pillars of the complex treatment of biofilm-infected wounds (Alves et al., 2021). It led to an increase in newly introduced or re-introduced antiseptic products intended to fight against wound biofilms. Examples of modern antiseptics are polyhexanide, octenidine dihydrochloride or povidone-iodine (Kramer et al., 2018; Eggers, 2019). On the other hand, such previously commonly used antiseptic as chlorhexidine was excluded from the guidelines protocols, mostly due to the increasing resistance (observed among others in microorganisms responsible for wound infections), including the development of cross-resistance against antibiotics (Kampf, 2016; Kramer et al., 2018). The growing frequency of chronic wound infections correlates the loss of patient’s health or even their death, as well as with the substantial financial cost for healthcare systems (Sen, 2019). Therefore, the search for new, efficient antiseptic agents is considered the clinical need of the highest importance. One of the types of antiseptics, presently re-introduced to wound treatment are the hypochlorites, including sodium hypochlorite (NaClO) and hypochlorous acid (HClO) alone or in combination with each other (Rembe et al., 2020). Significantly, the antiseptics containing a low content of substances as mentioned above (4-8 ppm) are presently claimed to display low cytotoxicity against wound cells (e.g., fibroblasts, keratinocytes) and to show particularly high antimicrobial activity (Esin et al., 2022). Nevertheless, these claims are derived mostly from past reports, part of which concern formulations of highly concentrated hypochlorites/hypochlorous acid or formulations supplemented with additional factor (or factors) of antibacterial activity (Severing et al., 2019). In turn, more recent reports show that however agents containing low concentrations of chlorine-based/releasing agents display a lack or no cytotoxicity, they, at the same time, indicate a low level of antimicrobial activity (Krasowski et al., 2021; Severing et al., 2022). Because the provision of an appropriate antiseptic agent to the patient suffering from the infected chronic wound is of pivotal meaning regarding the chances of therapeutic success, the present study aimed to investigate the antimicrobial/antibiofilm potential of two low-concentrated chlorine-based and releasing solutions, confronted with the antiseptic agent polyhexanide of acknowledged antimicrobial activity, applied in the character of control setting. To provide the data of a high usability, the analyses were performed not only using both reference and clinical strains of *Pseudomonas aeruginosa*, *Staphylococcus aureus*, and *Candida albicans* (to consider the differences between Gram-negative, Gram-positive bacteria and yeast; as well as intra-species variability), but also using the spectrum of *in vitro* models and different methodological approaches.

## 2 Materials and methods

### 2.1 Microorganisms

The research was carried out on bacterial and fungal strains from Collection of The Department of Pharmaceutical Microbiology and Parasitology, Medical University of Wroclaw, Poland. The pathogens included 25 clinical strains of methicillin-resistant *Staphylococcus aureus* (MRSA) and reference ATCC 33591 strain; 25 clinical strains of *Pseudomonas aeruginosa* and reference ATCC 27853 strain; 25 clinical *Candida albicans* strains and ATCC 10231 strain. All clinical strains, deposited in the collection of Pharmaceutical Microbiology and Parasitology Department, were isolated from infected chronic wounds of various etiology during routine microbiological diagnostics. The permission to use these strains in scientific projects conducted by the Department of Pharmaceutical Microbiology and Parasitology was granted by Bioethics Committee of Wroclaw Medical University, Poland; the permission protocol number # 8/2016. For the production of bacterial cellulose in the Cellulose-Based Biofilm model, the reference (German Collection of Microorganisms and Cell Cultures DSM 46604) *Komagateibacter xylinus* was applied.

### 2.2 Antiseptics

The liquid antiseptics applied in the study were under brand names:

- Prontosan Wound Irrigation Solution (later referred to as the “P”, B. Braun Medical AG, Melsungen Germany) containing polyhexamethylene biguanide (0.1%), undecylenamidopropyl betaine (0.1%), and purified water.
- Granudacyn Wound Irrigation Solution (later referred to “G”, Molnycke, Goteburg, Sweden) containing water, sodium chloride, hypochlorous acid (0.005%), sodium hypochlorite (0.005%),
- Microdacyn 60 Wound Care Solution (later referred to “M”, Sonoma Pharmaceuticals, Inc, Petaluma, CA) containing super-oxidized water, sodium chloride, hypochlorous acid (0.004%), sodium hypochlorite (0.004%).

### 2.3 Assessment of the ability of analyzed strains to form biofilm

#### 2.3.1 Assessment of formed biofilm biomass using a crystal violet staining

The bacterial and fungal suspensions of 0.5 McF (McFarland turbidity scale) density (measured using Densitomat II, BioMerieux, Warsaw, Poland) in 0.9% NaCl (Stanlab, Lublin, Poland) were prepared from the fresh, 24-hours cultures in TSB (Tryptic Soy Broth, Biomaxima, Lublin, Poland). The suspension of each strain was diluted 1000 times and portioned into 6 wells of a 96-wells plates (VWR, Radnor, PA, USA) in a volume of 100 µL per well. Next, the plates were incubated in the stationary conditions for 24 h at 37°C. Afterwards, the medium was gently removed and the plate was dried for 10 min at 37°C. Next, 100 µL of 20% aqueous solution of crystal violet (v/v) (Aqua-med, Lodz, Poland) was added to each well and left for 10 min at room temperature. Subsequently, the staining solution was removed, the wells were rinsed with 100 µL 0.9% NaCl twice and the drying process was repeated. To dissolve the stained biomass, 100 µL of 30% acetic acid (v/v) (Chempur, Piekary Slaskie, Poland) was added to each well and the plate was being shaken for 30 min at 450 rpm (Schuttler MTS-4, IKA, Königswinter, Germany) at room temperature. The color solutions were transferred to a fresh 96-wells plate and the absorbance was measured at wavelength 550 nm using a MultiScan Go Spectrophotometer (Thermo Fischer Scientific, Waltham, MA, USA).

#### 2.3.2 Assessment of the level of metabolic activity of analyzed biofilms using a resazurin assay

The biofilm was formed under the conditions described in section 2.3.1. Briefly, the suspensions at density 0.5 McF (McFarland turbidity scale) (Densitomat II, BioMerieux, Warsaw, Poland) in 0.9% NaCl (Stanlab, Lublin, Poland) were prepared and diluted 1000 times from the overnight cultures in TSB (Tryptic Soy Broth, Biomaxima, Lublin, Poland). 100 µL of inoculation was added to 6 wells in a 96-wells test plates (VWR, Radnor, PA, USA) and the plates were incubated for 24 h at 37°C. After the incubation time, 10 µL of 0.1% resazurin solution (Acros Organics, Geel, Belgium) in TSB was added to each well and the plates were incubated for 1 h, 2 h, and 3 h at 37°C. The colored medium was transferred to a new plate and the absorbance was measured at wavelengths 570 nm and 600 nm (MultiScan Go Spectrophotometer, Thermo Fischer Scientific, Waltham, MA, USA). The viability of cells was calculated by subtracting the absorbance value at 600 nm from the absorbance value obtained at 570 nm wavelength. Appropriate incubation times for tested species were determined based on the time the metabolic activity was visible, but the samples did not discolor and were as follows: *S*. *aureus* 1 h, *P. aeruginosa* 3 h, *C. albicans* 3 h.

### 2.4 Assessment of the antimicrobial/antibiofilm activity of antiseptics

#### 2.4.1 Minimal inhibitory concentration and minimal bactericidal/fungicidal concentration of P, G, M antiseptics using micro-titer plate assay

The geometric dilutions of tested substances (P, G, M) were prepared. For this purpose, 100 µL of TSB (Tryptic Soy Broth, Biomaxima, Lublin, Poland) was added to the 1-9 wells in one row of a 96-wells test plate (VWR, Radnor, PA, USA). Next, 200 µL of the P, G or M was added to the 10th well of the plate and diluted geometrically toward the first well. Simultaneously, microbial suspensions were prepared by diluting 0.5 McF density cultures (McFarland turbidity scale) (Densitomat II, BioMerieux, Warsaw, Poland) 1000 times in TSB. 100 µL of freshly prepared suspensions were added to the wells containing different concentration of substances. The control of microbial growth was the bacterial/fungal suspension in medium without test substance. The control of sterility was provided by medium without test substance and microbial suspension. Next, the absorbance was measured at 580 nm by the MultiScan Go Spectrophotometer, and the plate was incubated with shaking at 450 rpm for 24 hours at 37°C. The following day, the absorbance at wavelength 580 nm was measured again. No difference between two measurements indicated that the concentration in particular well with a substance concentration is considered the minimal inhibitory concentration (MIC). To confirm the MIC value, 20 µL of 0.1% resazurin in TSB was added to each well and incubated for 1 h (*S. aureus* strains) or 3 h (*P. aeruginosa* and *C. albicans* strains) at 37°C with shaking. The unequal incubation times were a result of different rate of metabolic activity between species assessed by the described method. The MIC value was determined visually first, on the basis of the color change of the medium from blue to the pink one. The colored medium was transferred to the fresh 96-well plate and its absorbance was measured at two wavelengths 570 nm and 600 nm by the MultiScan Go Spectrophotometer. The metabolic activity was calculated by subtracting the absorbance measured at 600 nm from the absorbance value measured at 570 nm and the results were used as a confirmation of a visual read. To determine the minimal bactericidal/fungicidal concentration (MBC), the bacterial/fungal suspension in a well with MIC and four neighbored wells with no metabolic activity were transferred to 2 ml of TSB in a 24-wells plate and incubated for 24 hours at 37°C. MBC was determined in the first well with no visual bacterial/fungal growth in the well. Three technical repetitions were performed for each strain and substance. For the cases in which the concentrations differed in one serial dilution, the higher concentration was chosen.

#### 2.4.2 Minimal biofilm eradication concentration and minimal bactericidal/fungicidal concentration against biofilm of P, G, M antiseptics using a micro-titer plate assay

Bacterial/fungal suspensions at density 0.5 McF (McFarland turbidity scale) (Densitomat II, BioMerieux, Warsaw, Poland) in 0.9% NaCl (Stanlab, Lublin, Poland) were prepared from fresh, 24-hours cultures in TSB (Tryptic Soy Broth, Biomaxima, Lublin, Poland). The suspensions were diluted 1000 times in TSB and portioned into 11 wells in a row in a 96-wells test plate (VWR, Radnor, PA, USA) of 100 µL to each. Next, 100 µL of TSB was added to each well. The plate was incubated for 24 hours at 37°C under stationary conditions. The next day, the medium above the biofilm was gently removed and geometric dilutions of tested substances were prepared. Briefly, 200 µL of TSB was added to the first 9 wells in a 96-wells plate. 400 µL of the tested substance was added to the 10th well, and transferred to the wells containing TSB to dilute it geometrically. Next, the geometric dilution of tested substances was transferred to the plate with biofilm and incubated for 24 h at 37°C in stationary conditions. The control setting of sterility and bacterial/fungal growth were provided as described in the section 2.5.1 After incubation, 20 µL of 0.1% resazurin (Acros Organics, Geel, Belgium) in TSB was added to each well and incubated for 1 hour (*S. aureus* strains) or 3 hours (*P. aeruginosa* and *C. albicans* strains) at 37°C. The MBEC was determined on the basis of the medium color change. To confirm the visual readout, the colored medium was transferred to the new 96-wells test plate and the absorbance was measured at the wavelengths 570 nm and 600 nm using the MultiScan Go Spectrophotometer (Thermo Fischer Scientific, Waltham, MA, USA). The viability was assessed by subtracting the absorbance values obtained at 600 nm from absorbance values obtained at 570 nm. Next, the content of the MBEC well and four neighbored wells without color change were transferred to 2 mL of TSB in a 24-wells plate (VWR, Radnor, PA, USA) to assess the minimal bactericidal/fungicidal concentration against biofilm (MBC-B). This value was determined in the first well with no visual growth (change in turbidity). Three technical repetitions of this assay were performed. For the cases in which the concentrations differed in one serial dilution, the higher concentration was chosen.

#### 2.4.3 Assessment of antibiofilm activity of P, G and M antiseptics using biofilm-oriented antiseptics test

The BOAT was performed following the protocol devised previously (Junka et al., 2014). To perform the test, four strains of each species with the highest MBEC (assessed by the method described in the penultimate section) and all reference strains were chosen. Fresh, 24-hours bacterial/fungal cultures in TSB (Biomaxima, Lublin, Poland) were suspended in 0.9% NaCl (Stanlab, Lublin, Poland) to achieve density at 0.5 McF, and diluted 1000 times. 100 µL of such prepared suspensions were added to wells in a 96-wells plate and incubated for 24 hours at 37°C under stationary conditions. Next, the medium was gently removed and 100 µL of the test substance was added to the well with adhered biofilm. The contact times were 15 min, 30 min, 1 hour and 24 hours at 37°C. After exposure, the substance was gently removed and 100 µL of the neutralizer peptone saline water (1 g/L casein, 8.5 g/L NaCl) was added for 5 min at room temperature analogically as it was performed in our earlier works (Junka et al., 2014; Krasowski et al., 2021). The peptone saline water was remove and 100 µL of 1% tetrazolium chloride salt (TTC, 2,3,5-triphenyl-2H-tetrazolium chloride, PanReac AppliChem, Darmstadt, Germany) in TSB, and incubated for 2 hours at 37°C. A red color indicated metabolic activity in the well and was marked as “+”, no viability (no color change) was marked as “-”. The growth control was provided by medium in well with biofilm without a test substance. The control of sterility was provided by medium with substance without a biofilm. Then, the medium was removed and 100 µL of solution of 90% methanol (Chempur, Piekary Slaskie, Poland) and 10% acetic acid was added to each well to dissolve red formazan crystals. The plate was being shaken for 30 min at room temperature and afterward, the absorbance was measured at wavelength 490 nm by the MultiScan Go Spectrophotometer. The experiment was performed at six repetitions for each strain and substance. The viability (V) was calculated with the following Equation (1):


$$V\;\left[\%\right]=100\%\;\times \frac{Abs_{GC}}{Abs_{T}}$$

where Abs_GC_ stands for an average value of absorbance values obtained for growth control, and Abs_T_ stands for an average of absorbance values obtained for wells treated with substance. Two strains of *Pseudomonas aeruginosa* scantly metabolized TTC. To evaluate their susceptibility to tested substances, the well content (after the contact time) was seeded on the Müller-Hinton agar (Biomaxima, Lublin, Poland). The cultures were incubated for 24 h at 37°C. Three technical repetitions for each substance and contact time were performed. The growth control setting was also provided for each contact time.

#### 2.4.4 Assessment of antibiofilm activity of P, G and M antiseptics using cellulose-based biofilm model

The suspension of 2x10^5^ CFU/mL of *Komagateibacter xylinus* obtained from a 7-day culture, was introduced to the self-prepared Herstin–Schramm medium containing the following ingredients: glucose (2% w/v; POCH, Gliwice, Poland), yeast extract (0.5% w/v; Graso, Starogard Gdanski, Poland), bacto-pepton (0.5% w/v; Graso, Starogard Gdanski, Poland), citric acid (0.115% w/v; POCH, Gliwice, Poland), Na_2_HPO_4_ (0.27% w/v; POCH, Gliwice, Poland), MgSO_4_x7H_2_O (0.05% w/v; POCH, Gliwice, Poland), bacteriological agar (2% w/v; Graso, Starogard Gdanski, Poland) and ethanol (1% v/v; POCH, Gliwice, Poland). The bacterial cellulose biosynthesis was carried out in 24-well plates. The obtained bacterial cellulose carriers (BC) in the form of 18-mm diameter cylinders were subjected to the alkaline lysis (to remove *K. xylinus* cells) and thoroughly rinsed with sterile water until pH value obtained the neutral value. The 10 mL of the sterile DMEM (Biowest, Riverside, MO, USA) medium was introduced to BC carriers and left for 48 hours at 4°C. Next, the suspension containing 10^5^ cells/mL of murine fibroblasts L929 (ATCC, Manassas, VA, USA) in the DMEM medium was settled on the BC. The fibroblast proliferation was assessed by means of a routine tetrazolium test every 24 hours for 7 subsequent days. Next, 2 mL of 10^5^ CFU/mL of *C. albicans*, *S. aureus*, or *P. aeruginosa* was introduced on the BC carrier containing a fibroblast layer. Half of the medium was replaced with the fresh one every 24 hours. The viability and proliferation of the microbial cells were evaluated using quantitative culturing on the appropriate solid agar plates (Müller-Hinton agar for bacteria and Sabouraud agar for yeast (Biomaxima, Lublin, Poland)) and tetrazolium test every 24 hours for 3 days. The 24 h co-culture of microbes and fibroblasts was chosen as a model for antiseptic application, similarly as we have shown it earlier (Czajkowska et al., 2021). Such prepared biofilm grown on the fibroblast-containing BC carrier was exposed to 2 mL of P, G or M antiseptics for 1 hour of contact time. Next, the carriers containing remaining biofilm were transferred to 10 mL of neutralizer (peptone saline water (1 g/L casein, 8.5 g/L NaCl) for 5 min. After this time, the BCs were introduced to 2 mL of BHI medium (Biomaxima, Lublin, Poland) containing 1% TTC and left for 2h. After incubation, the medium was removed, and the BCs were rinsed again with 0.9% NaCl. Afterwards, 1 mL of a solution containing ethanol: acetic acid 90:10 (v/v), respectively, (POCH, Gliwice, Poland) was introduced to the BCs to extract the formazan and subjected to mechanical shaking for 15 min. After shaking, the formazan solution was transferred to fresh 96-well plates and quantified at a wavelength of 490 nm by the MultiScan Go Spectrophotometer (Thermo Fischer Scientific, Waltham, MA, USA). To assess the percentage of remaining biofilm-forming cells in the fibroblast-containing BCs samples in comparison to the untreated (control) setting. The BCs with pre-formed biofilm treated with 0.9% NaCl instead of P, G, or M substances served as a control of biofilm growth. The biofilm eradication (E) was calculated with the following Equation (2):


$$\text{E}\;\left[\%\right]=100\%-\frac{Abs_{C}}{Abs_{T}}x100$$

where Abs_C_ stands for an average value of absorbance values obtained for control, and Abs_T_ stands for an average of absorbance values obtained for test sample.

#### 2.4.5 The analysis of P, G or M activity against biofilms formed in the CDC bioreactor

The P, G or M activity against 24 h-old biofilms was analyzed in the CDC bioreactor (CTG, Bozeman, Montana, USA). The liquid cultures were diluted in TSB medium to reach the 10^8^ CFU/mL, introduced to the CDC bioreactor, and subjected to incubation for 24 h/37°C/120 rpm. Next, coupons were rinsed with 0.9% NaCl and placed into the 6-wells plate (VWR, Radnor, PA, USA). The 4 mL of P, G or M were introduced to the plate’s wells. The setting to which 4 mL of 0.9% NaCl instead of antiseptic was introduced, served as growth control of the experiment. The setting was incubated for 37°C for 24 hours. After the exposure, the antiseptics were removed and the neutralizer peptone saline water (1 g/L casein, 8.5 g/L NaCl) was introduced. After neutralization, the coupons were subjected to vortex mixing in 5 mL of 0.1% saponin (Merck, Darmstadt, Germany) to detach the biofilms and disintegrate the cellular aggregates. In the next steps, the quantitative culturing of serial dilutions of obtained suspension was performed on the appropriate agar plates (Müller-Hinton agar for bacteria and Sabouraud agar for the yeast (Biomaxima, Lublin, Poland)). The plates were incubated for 24 hours at 37°C. After incubation, the number of colonies was counted and their number was compared to the setting where 0.9% NaCl instead of P, G or M antiseptics was applied.

#### 2.4.6 The analysis of P, G or M activity against biofilms in flow conditions generated with the bioflux device

In the microfluidic version, assessing biofilm formation, the Bioflux 1000 system (Fluxion, San Francisco, CA, USA) was applied. In the first stage of the experiment, the microfluidic channels were flushed from inlets to outlets with TSB medium with a speed of 10 dyne/cm^2^ for 10 s. Thereafter, 0.1 mL of microbial solutions in TSB medium (OD_600_ = 1.0) was put into each outlet wells. The flow of microbial solutions was turned on towards the outlet to inlet wells with 5 dyne/cm^2^ for 5 s. The solutions were left for 1-hour incubation at 37°C to allow microbes to adhere to the microcapillaries’ surface. Next, 0.9 mL of TSB medium was added to each of the inlet wells and the medium flow was turned on from inlet to outlet wells with an intensity of 0.5 dyne/cm^2^ for one day and 37°C. After 24-h culture, both the inlet and outlet wells were drained. 0.5 mL of solution, being one of the tested P, G or M antiseptics with TSB medium in a 1:1 ratio, was added to the inlet wells and directed for a 30 min medium flow with a rate of 1.5 dyne/cm^2^ (inlet to outlet). For positive controls, 0.5 mL of TSB medium was used. Following, inlet wells were again emptied and filled with 0.1 mL of a saline solution with 0.3 μL of the fluorophores SYTO 9 and 0.3 μL propidium iodide (both from FilmTracer™ LIVE/DEAD™ Biofilm Viability Kit; ThermoFisher Scientific, Waltham, MA, USA) for *S. aureus* and *P. aeruginosa* or 0.1 mL of a saline solution with 0.15 μL of calcofluor white (18909-100ML-F; Sigma Aldrich, St. Louis, MO, USA) and 0.3 μL propidium iodide for *C. albicans*. These solutions were passed through microcapillaries for 1 hour in the outlet direction. Photographs of microbial biofilms were taken with an inverted microscope (Carl Zeiss GmbH, Jena, Germany). The degree of biofilm development interpreted based on the microcapillaries’ coverage, and the ratios of green/red fluorescence constituting information about the viability of biofilms were calculated using the ImageJ software version 8 (National Institutes of Health, Bethesda, MD, USA). In case of *C. albicans* biofilm which did not stain using L/D methodology, the degree of biofilm development was also calculated using ImageJ software but using ratio of surface covered with biofilm vs surface non-covered with biofilm.

### 2.5 Confocal microscopy examination of chosen biofilms

The biofilms were dyed with FilmTracer™ LIVE/DEAD™ Biofilm Viability Kit (Thermo Fischer Scientific, Waltham, MA, USA) according to the manufacturer’s instruction. The subsequent procedures related to cells visualization were performed analogically to those presented in the earlier work (Krasowski et al., 2021).

### 2.6 Statistical analyses

Calculations were performed using GraphPad Prism (Version 8.0.1; GraphPad Software Inc., La Jolla, CA, USA, www.graphpad.com (accessed on 25 March 2022)). The Shapiro-Wilk and Levene tests assessed the normality distribution and variance homogeneity. Non-parametric test, like ANOVA Kruskal-Wallis with *post hoc* Dunn’s analysis, was applied to compare the efficacy of tested substances. The differences with a significance level of p< 0.05 were considered significant.

## 3 Results

**Figure 1:**
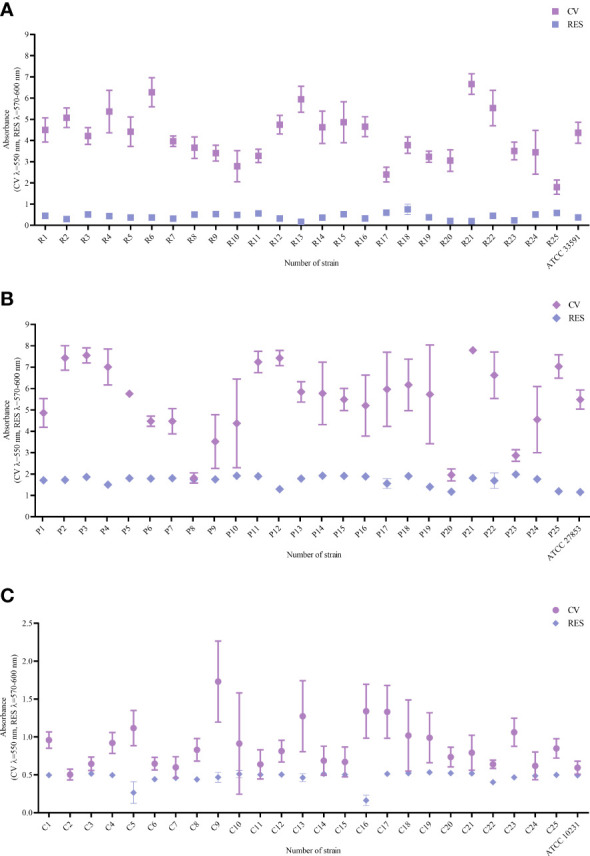
Ability to form biofilm assessed with the crystal violet method CV and metabolic activity assessed with the resazurin staining RES of **(A)** *Staphylococcus aureus* strains R1-R25 and ATCC 33591, **(B)** *Pseudomonas aeruginosa* strains P1-P25 and ATCC 27835, **(C)** *Candida albicans* strains C1-C25 and ATCC 10231. Average values and standard deviations are marked; ATCC – American Type Culture Collection.

**Figure 2:**
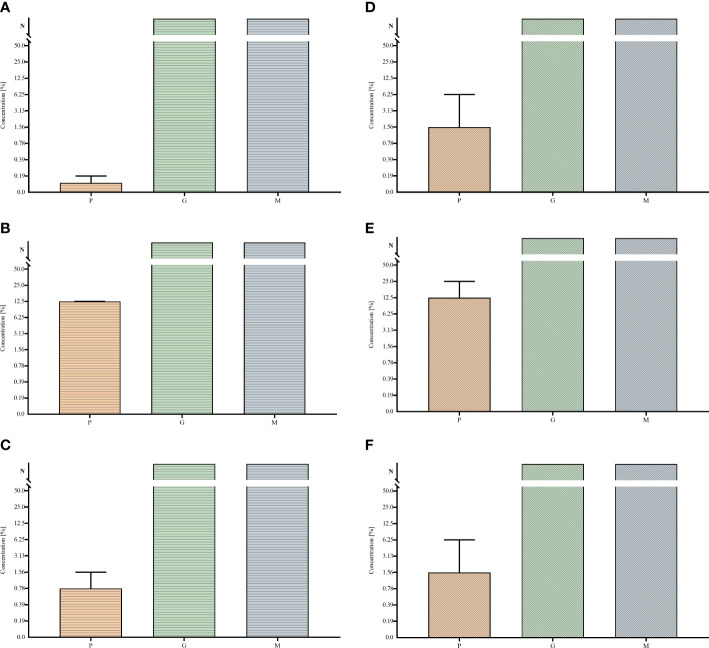
Minimal inhibitory concentrations MIC **(A–C)** and minimal bactericidal/fungicidal concentrations MBC **(D–F)** of polyhexanide P and hypochlorite-containing solutions G, M towards *Staphylococcus aureus* **(A, D)**, *Pseudomonas aeruginosa* **(B, E)** and *Candida albicans* **(C, F)** strains. Median and range are presented. N – non-measurable within tested concentrations range of antiseptics.

**Figure 3:**
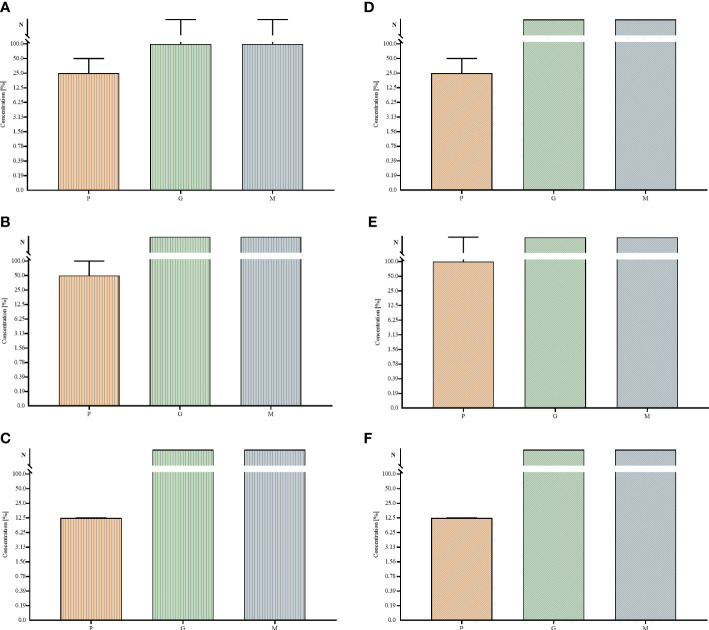
Minimal biofilm eradication concentrations MBEC **(A–C)** and minimal bactericidal/fungicidal concentrations for biofilm MBC-B **(D–F)** of polyhexanide P and hypochlorite-containing solutions G, M against *Staphylococcus aureus* **(A, D)**, *Pseudomonas aeruginosa* **(B, E)** and *Candida albicans* **(C, F)** strains. Median and range are presented. N – non-measurable within tested concentrations range of antiseptics.

In the first line of investigation, the ability of tested strains to form biofilm in the applied setting was measured (**Figure 1**). For this purpose, the amount of biofilm biomass and the metabolic activity of the strains were assayed using colorimetric methods, referred to as the crystal violet and resazurin staining, respectively. All analyzed strains were able to form the biofilm biomass and displayed the measurable level of metabolic activity in the applied setting, nevertheless obtained results showed high differences (of both intra- and inter-species character) regarding both of the analyzed features. The specific strains differed in their biofilm biomass level (understood as the cells and the extracellular matrix together) up to 2 times and even up to 4 times regarding the differences in levels of their metabolic activity. Moreover, all possible combinations between values of analyzed parameters were recorded, among tested strains, i.e. high (among tested strains) metabolic activity and low (among tested strains) level of biofilm biomass (e.g. *P. aeruginosa* strain P3); low metabolic activity and high biomass level (e.g., *P. aeruginosa* strain P23), low metabolic activity and low biomass level (e.g., *P. aeruginosa* strain P20) or high metabolic activity and high biomass level (e.g., *P. aeruginosa* strain P11). The analogical relationships between the two analyzed parameters were also observed in the case of two other species tested, *S. aureus* and *C. albicans*. Such an observation shows the high intra-species variability regarding the ability of biofilm formation in the applied experimental setting, resulting, as shown in **Figure 2**, in differentiated levels of the antimicrobial activity of P, G, M against tested pathogens. Such a variability could be observed in the most distinct manner regarding the P antiseptic. This compound was of significantly higher efficacy against all tested pathogens than G and M antiseptics, nevertheless, relatively high range of MIC values were observed. A similar phenomenon was not stated in the case of G and M antiseptics because their antimicrobial activity, in this particular experimental setting, did not reach the level of MIC or MBC value within tested ranges of concentrations. The data presented in **Figure 2** refers to the suspended microbial cells (the planktonic cells), while data presented in **Figure 3** refers to the results of antimicrobial activity of P, G and M, against biofilm (cultured in the same manner as biofilm whose biomass level and metabolic activity were assessed and presented in **Figure 1**). Also, in the case of the level of biofilm eradication, the P antiseptic displayed significantly higher activity compared with G and M antiseptics (**Figure 3**).

In the next investigation line, the biofilm oriented antiseptic test (BOAT) was performed to assess the antimicrobial potential of P, G and M antiseptics in 15, 30, 60 minutes and within 24 hours of contact time. By means of BOAT not only more relevant and clinically oriented contact times may be applied (comparing to the MIC/MBEC assay method presented in **Figure 2** and **Figure 3**), but also the undiluted concentration of antiseptic (referred to as 100% or “working solution”) may be introduced to the experimental setting. Results presented in **Table 1**, show the significant superiority of P antiseptic compared to G and M antiseptic, nevertheless also M and G antiseptic showed the ability to eradicate the biofilm of particular strains of *S. aureus* and *P. aeruginosa* within 24-hour contact time. On the other hand, the G and M antiseptics were not able to eradicate any of the tested *C. albicans* biofilm-formed strains within this time period. In turn, P antiseptic displayed the highest activity against *P. aeruginosa* than *S. aureus* biofilm, whereas the complete eradication of *C. albicans* biofilm was observed in 1 h and 24 h contact times only (regardless the biofilm-forming strains) but not in shorter (15’, 30’) contact times. For samples in which microbial growth was detected, the metabolic activity of bacteria and fungi was calculated compared to growth controls using equation 1. The metabolic activity of *S. aureus* was reduced by 67%-97% after exposure to P at 15 min and 30 min contact time. In contrast, G and M reduced bacterial viability mostly by 5%. The effectiveness of P against *C. albicans* biofilm was comparable between 15 min and 30 min and reduced the metabolic activity by 59% to 83%. It has to be highlighted that some of the staphylococcal and fungal strains showed increased activity in the presence of G and M compared to the growth control in every time spot. Contrarily, the viability of pseudomonal strains was higher after exposure to P than in the case of applying G or M.

**Table 1:** Antimicrobial efficiency of polyhexanide P and hypochlorite-containing solutions G, M against *Staphylococcus aureus*, *Pseudomonas aeruginosa* and *Candida albicans* biofilm in four contact times (15 min., 30 min., 1 h, 24 h) achieved by means of the biofilm-oriented antiseptics test BOAT.

STRAIN No.		P				G				M			
		15’	30’	1h	24h	15’	30’	1h	24h	15’	30’	1h	24h
*S. aureus*	15	+	+	–	–	+	+	+	–	+	+	+	+
*S. aureus*	21	+	+	+	–	+	+	+	–	+	+	+	–
*S. aureus*	22	+	+	+	–	+	+	+	+	+	+	+	+
*S. aureus*	25	+	–	–	–	+	+	+	–	+	+	+	–
*S. aureus*	ATCC 33591	+	+	–	–	+	+	+	–	+	+	+	+
*P. aeruginosa*	5	+	+	–	–	+	+	+	+	+	+	+	+
*P. aeruginosa*	9	+	–	–	–	+	+	+	+	+	+	+	+
*P. aeruginosa*	19	+	–	–	–	+	+	+	–	+	+	+	–
*P. aeruginosa*	22	+	+	–	–	+	+	+	–	+	+	+	–
*P. aeruginosa*	ATCC 27853	+	–	–	–	+	+	+	+	+	+	+	+
*C. albicans*	9	+	+	–	–	+	+	+	+	+	+	+	+
*C. albicans*	10	+	+	–	–	+	+	+	+	+	+	+	+
*C. albicans*	11	+	+	–	–	+	+	+	+	+	+	+	+
*C. albicans*	14	+	+	–	–	+	+	+	+	+	+	+	+
*C. albicans*	ATCC 10231	+	+	–	–	+	+	+	+	+	+	+	+

Metabolically active strains are marked as ‘+’, no visually viable strains are marked as ‘-’. ATCC – American Type Culture Collection.

**Figure 4:**
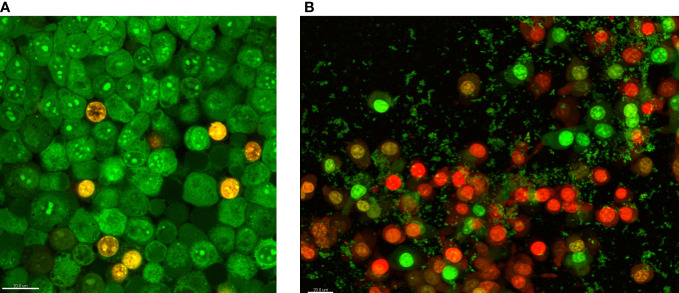
The fibroblasts’ growth on the bacterial cellulose surface **(A)** and fibroblast co-cultured with staphylococcal biofilm (small, green cellular aggregates) in the cellulose-based biofilm CBB model **(B)**. The eukaryotic and bacterial cells are dyed with a mixture of SYTO-9 and Propidium iodine. The color red/orange indicates dead or damaged cells, while green indicates live, intact cells. The scale bar in the left corner of images is 20 µm.

**Figure 5:**
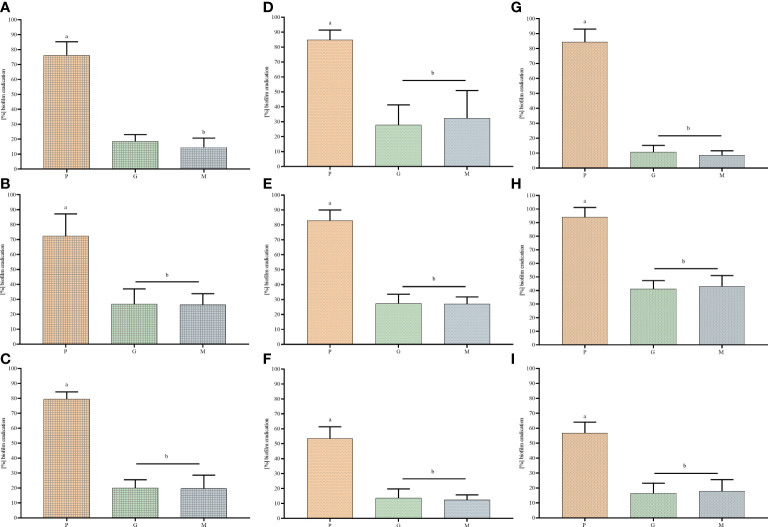
The biofilm eradication of *Staphylococcus aureus* **(A, D, G)**, *Pseudomonas aeruginosa* **(B, E, H)** and *Candida albicans* **(C, F, I)** after treatment with polyhexanide P and hypochlorite-containing solutions G and M, measured in cellulose-based biofilm CBB model **(A–C)**, CDC bioreactor model **(D–F)** and the Bioflux device model **(G–I)**. Pairs of letters (a/b) refer to the differences being statistically significant (p< 0.05).

The third experimental model utilized porous, three-dimensional cellulosic polymer (BC carrier) as a scaffold on which fibroblast monolayer was developed (**Figure 4**). The microbial biofilm was introduced to such a setting and cultured with the use of a medium designed for the development of eukaryotic cells (with no antibiotics added). Such a model mimics the wound environment to a greater extent than previously described microtiter assays where a flat polystyrene surface and routine microbiological medium are used. Results of CBB model, presented in **Figure 5**, show a higher level of biofilm eradication after the use of G and M antiseptics than was observed in standard MBEC evaluation, presented in **Figure 3**. Nevertheless, the eradication level was still moderate, reaching ca. 20% with regard to *S. aureus* and *C. albicans* and about 30% concerning *P. aeruginosa*. The P antiseptic was significantly more efficient (p< 0.05), than G and M antiseptic, reaching the eradication level of about 80% with regard to *S. aureus* and *C. albicans* biofilms and about 70% with regard to the *P. aeruginosa* biofilms. In the case of the later pathogen, the highest range of eradication values were observed, regardless the antiseptic applied.

The results obtained in another model, namely the CDC bioreactor, showed lower level of biofilm eradication compared to the cellulose-based biofilm model, regardless of the strains applied (**Figure 5**). Nevertheless, the same trend was observed as in case of earlier-presented *in vitro* models, i.e. the biofilm eradication being result of P antiseptic introduction was significantly (p< 0.05) higher than biofilm eradication being result of G or M introduction. These two hypochlorite-containing antiseptics displayed higher activity against *P. aeruginosa* (than against *S. aureus* or *C. albicans*), nevertheless the level of biofilm eradication did not cross the value of 40% regardless of any *P. aeruginosa* strain.

**Figure 6:**
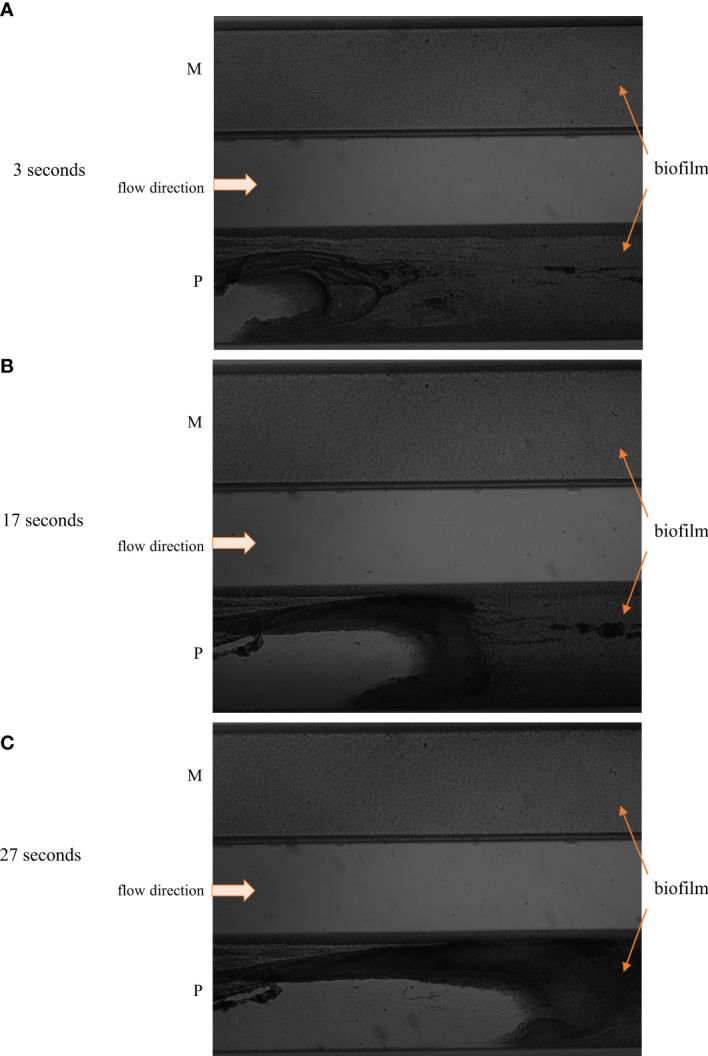
The image of staphylococcal biofilm subjected to polyhexanide P and hypochlorite-containing solution M in the flow conditions in the short contact time 3 sec. **(A)**, 17 sec. **(B)**, 27 sec. **(C)**. The staphylococcal biofilm is indicated by the orange arrows, while the flow direction is indicated by the white arrow. The progress of staphylococcal biofilm detachment is seen in the setting where P antiseptic was applied (left part of the “P” flow cell), while changes in staphylococcal biofilm subjected to M antiseptic are not visible in the bright field.

**Figure 7:**
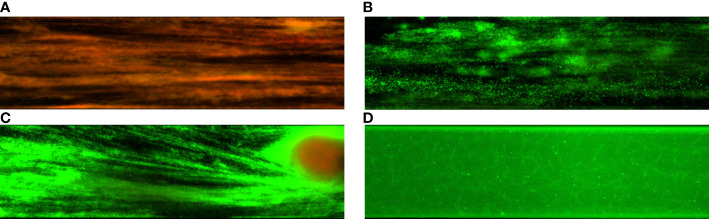
Comparison of staphylococcal biofilms exposed to polyhexanide P **(A)** and hypochlorite-containing solutions G **(B)** and M **(C)**. The control setting **(D)** was provided by the staphylococcal biofilm rinsed with saline.

Finally, the antibiofilm activity of P, G and M antiseptics was scrutinized using a Bioflux device measuring not only the antimicrobial potential of antiseptic (understood here as the ability to kill biofilm-forming cells) but also the ability of biofilm to resist the force of shear flow (as shown in **Figure 6** and **Figure 7**). The eradication of pseudomonal biofilm by means of G and M antiseptics was the highest compared to data obtained by means of all previously presented *in vitro* models, nevertheless, the value of the parameter discussed did not exceed 50% (**Figure 5**). In the case of staphylococcal and *Candida* biofilm, values of eradication oscillated between 10-20%, regardless of whether G or M antiseptic was applied. The P antiseptic displayed a significantly higher ability to eradicate the biofilm (p< 0.05), regardless of the species forming this structure, comparing to M or G antiseptics. Nevertheless, the ability of P antiseptic to eradicate *C. albicans* biofilm was significantly lower (p< 0.05) comparing to the ability of this compound to eradicate biofilms formed by *S. aureus* or *P. aeruginosa*.

## 4 Discussion

In developed countries, the occurrence rate of a chronic wound is considered to be c.a. 2% of the total population. The most recent data indicate that 75-80% of such skin and soft tissue discontinuities is colonized/infected by microbial biofilms (Thaarup et al., 2022). Nevertheless, the aforementioned percentage may be underestimated, mostly due to difficulties related to the accurate diagnostics of biofilm in the wounds. Although wound biofilms have been thoroughly investigated for at least 20 years, recent reports show that the factual significance of these structures and their functionalities in the process of wound healing/delay is still to be fully elucidated (Probst et al., 2022). The advances in the understanding wound biofilms are significantly disturbed due to the lack of reliable, quantitative methods of studying *in vivo* wound biofilms in real-time. The relatively high area of chronic wounds (and small size of microbial cells), presence of blood, exudate, pus and necrotic tissues, un-even distribution of biofilm aggregates in the wound (Malone et al., 2017), the chemical resemblance of biofilm matrix components to components of eukaryotic cell walls and eukaryotic matrix (Ciecholewska-Juśko et al., 2022), and last but not least high structural/functional adaptability of biofilm to the changing stimuli originated from human host (González et al., 2018), constitute the multi-variable, highly-complex system of human-pathogen interactions, which analysis is fraught with the high risk of drawing the flawed conclusions. Nevertheless, the presence of biofilms in wounds is thought to result in long-term infections and to be extremely difficult to eradicate (Bjarnsholt et al., 2008; Versey et al., 2021). Thus, the understanding of wound biofilms and the ways of their removal is a matter of paramount importance.

Therefore, a variety of *in vitro* tests was proposed – to reduce the number of variables and to provide insight into the possibilities of fighting them off with the use of antimicrobial substances (Stuermer et al., 2021; Vyas et al., 2022; Pham et al., 2023). Of note, certain *in vitro* wound biofilm models are performed on smooth, abiotic surfaces (which do not resemble the wound surface), and they lack also such relevant factors as flow or adequate medium resembling the wound fluid. Moreover, the number of studies performed with reference strains only (and not with the factual, wound-colonizing strains), neglecting the importance of inter-species variability (Woroszyło et al., 2021). These issues are factors that undoubtedly drift away the *in vitro* wound biofilms model from the factual wound biofilms and hinder drawing the appropriate conclusions from the performed analyses. On the other hand, our knowledge of factual wound biofilms is still extremely limited. Following this line of reasoning, the higher number of differentiated *in vitro* models (even if they do not fully reflect the wound environment) and microbial strains applied in particular *in vitro* research, the more obtained results should be considered reliable. Therefore, in the present work we applied 6 different *in vitro* tests and together 75 clinical and reference strains of 3 relevant (isolated from chronic wounds) species (*S. aureus*, *P. aeruginosa*, *C. albicans*). The aim of the study was to compare the efficacy of low hypochlorite-containing solutions vs. polyhexanide-containing antiseptic. The reason behind this agenda was the contradictory results concerning the antimicrobial (including antibiofilm) potential of these earlier antiseptics (Barrigah-Benissan et al., 2022; Severing et al., 2022). The inclusion of the polyhexanide-base antiseptic, served as the control setting of experiments because antimicrobial/antibiofilm potential of PHMB is generally accepted and recognized in the *in vitro* and clinical studies) (To et al., 2016; Kramer et al., 2018). The topic undertaken in this research is thus of pivotal meaning with regard to chronic wound infections, because locally-administered antiseptics represent a crucial part of modern algorithms of wound management (Kramer et al., 2018). Although the application of antiseptic alone is considered insufficient (in most cases) in the provision of chronic wound healing and closure, nevertheless, antiseptics combined with debridement and modern dressing application may translate into a biofilm eradication resulting in decrease in infection rate and more rapid advance of wound healing.

The preliminary tests, performed in this study, on the ability of strains to form biofilm (measured by assessment of a biofilm biomass and metabolic activity of biofilm cells, **Figure 1**) indicated the high intra- and inter-species differences in this regard. It stays in line with results presented by other research teams (Woroszyło et al., 2021) and indicates the necessity of inclusion of a broad spectrum of various strains for the preliminary tests, because the high ability to form biofilm translates into higher potential resistance of strains to antimicrobials. For the purposes of the current study, the sample of strains showing weak, moderate and high (among the tested) ability to form biofilm was chosen, providing a representative cross-section of the whole tested group of pathogens. Of note, the data obtained for *P. aeruginosa* strains displayed the highest standard deviations and such a phenomenon is most likely related also with still unsolved disadvantage of microplate testing of slime-forming species belonging to the Pseudomonadaceae family (Kragh et al., 2019).

The MIC and MBC assessments using standard micro-titer plate settings showed that hypochlorite-based antiseptics (G and M) were unable to eradicate (destroy) cells of tested pathogens (**Figure 2**). In this experimental model, the colorimetric measurement was confirmed by quantitative culturing cells on the agar plates. Therefore, the obtained results show the ability (or lack of ability) of the specific concentration of investigated antiseptics not only to stop the metabolic activity of cells but also to kill (or not to kill) the whole cellular population within the specific well of micro-titer well. In turn, the specific concentrations of P antiseptic within range of the working solution were able to kill investigated strains. The highest tolerance to P antiseptic was displayed by *P. aeruginosa* strains, nevertheless, the median MIC/MBC value for all tested strains oscillated within the range of 8 times diluted working solution of P antiseptic. Higher MIC/MBC values of P against *P. aeruginosa* strains (than against *S. aureus* and *C. albicans*) may be once again explained by the already mentioned fact of the presence of a slimy protective matrix of the earlier pathogen. Although the MIC/MBC assessment in the microtiter plate aims to test the planktonic (free, non-adhered) cells of pathogens, some reports indicate that the cellular community of Pseudomonadaceae (but also Enterobacterales) in such an experimental setting may consist of a mixed population of planktonic cells and aggregates of cells, which are adhered by the extracellular matrix not to the surface but to themselves (Alhede et al., 2011). When the standard microtiter assay was applied to measure the efficacy of P, G and M antiseptics against biofilms of tested pathogens, one may notice increase (up to eight times) of P antiseptic concentration necessary to eradicate the biofilm, which stays in line with the generally accepted phenomenon of higher tolerance of biofilm towards antimicrobials comparing to the level of tolerance of the planktonic cells (Lappin-Scott et al., 2014). In the case of G and M antiseptics, no MBEC value was recorded (the highest applied concentration of these both antiseptics was insufficient to eradicate the biofilm in this setting). Results of the Biofilm Oriented Antiseptics Test (**Table 1**) which revealed that all of the tested biofilms, exposed to G and M antiseptics were able to survive within shorter contact times (15 - 30 minutes and 1 hour), while the ability to eradicate part of the biofilms occurred only in the longest applied contact time (24 hours) with regard to the *S. aureus* and *P. aeruginosa* but not with regard to *C. albicans* strains. Of note, M antiseptic eradicated two times more *S. aureus* strains than G antiseptic in the longest contact time, while P antiseptic eradicated all tested strains of *S. aureus*, *P. aeruginosa* and *C. albicans* in this contact time. In turn, in 1h of contact time, P antiseptic was able to eradicate 86% of tested strains, while the level of eradication measured for 30 minutes was 20%. Such an observation is consistent with results presented by another research team (Severing et al., 2022), nevertheless one should remember that BOAT test (similar to microtiter plate MIC/MBEC assays) provides data on the ability of given antimicrobial to fully eradicate the pre-formed biofilm (Junka et al., 2014). Therefore, to get more insight into the factual activity of investigated antiseptics, another *in vitro* model was applied. Contrary to microtiter MIC/MBEC and BOAT assays, the cellulose-based biofilm model (CBB model) uses a polymeric, porous surface of cellulose covered with fibroblasts to imitate the environment of a wound bed (**Figure 4**). Moreover, the microbial biofilm is cultured using a medium for fibroblast cells instead of a traditional microbiological medium. The high impact of media resembling human body fluid on bacterial biofilms, resulting in their altered physiology and decreased tolerance to antimicrobials (antiseptics and antibiotics), was showed by another research team and us recently (Nielsen et al., 2018; Paleczny et al., 2021; Paleczny et al., 2022). The results obtained by means of CBB model confirmed the data obtained by means of earlier-described models (i.e. significantly higher activity of P antiseptic comparing to G and M antiseptics) (**Figure 5**). On the other hand, the application of CBB model allowed to indicate that both G and M antiseptics were able to partially remove biofilm of the tested pathogens from the cellulosic polymer. In turn, the antibiofilm activity of P antiseptic measured in this particular model was lower (oscillating between eradication levels equal to 70-90%) than in the case of models utilizing polystyrene surface together with the microbiological medium. Such an observation seems to confirm the impact of the surface applied in the tests. One may hypothesize that biofilm-forming microorganisms embedded within the un-even and porous cellulosic mesh are harder to be reached by penetrating antiseptic, than in the case of biofilm grown on the flat polystyrene surface (Gomes and Mergulhão, 2017).

The experiments performed in the CDC bioreactor confirmed the higher ability of P antiseptic to eradicate biofilm compared to G and M antiseptic (**Figure 5**). The levels of eradication were comparable to those obtained in the CBB model, i.e. lower than those presented for biofilm cultured on a polystyrene surface in static conditions. In turn, biofilm obtained by means of CDC was cultured in the environment providing centrifugal force and shear stress (**Figure 6** and **Figure 7**). Such conditions translate into increased viability of biofilm cells and biofilm’s density, which may explain the lower activity of tested antiseptics (Yang et al., 2019). The shear force is an even more essential factor in the last experimental model applied (Bioflux device) in which biofilms were formed in the flow conditions of 0.5 dyne/cm^2^/24 h and then subjected to the P, G or M antiseptic flowed with the force of 1.5 dyne/cm^2^. The results obtained in the Bioflux model correlated to a higher extent with the results obtained by means of CBB and CDC models than with microplate (MBEC) and BOAT models. Still, the P antiseptic acted significantly more effectively than G and M antiseptics. Interestingly, the *P. aeruginosa* biofilms were eradicated more efficiently than *S. aureus* biofilms regardless of the antiseptic applied. As already mentioned, *P. aeruginosa* biofilms consist of a slimy, cohesive matrix. It may be hypothesized thus, that by means of applied shear force, the overall higher amount of biofilm structure was de-attached, because initially de-attached fragments, being still linked to the remaining parts of biofilm structure, eventually drew them away. Such results and possible explanations of this phenomenon indicate the pivotal meaning of the de-attaching ability of antiseptics (often provided by various surfactants present in the antiseptic products) in the process of biofilm eradication.

In this work, the total 75 microbial strains, being causative factors of chronic wound infections, were scrutinized regarding their tolerance/sensitivity towards two hypochlorite-based agents compared to the polyhexanide-based antiseptic of acknowledged antimicrobial/antibiofilm activity. The performed analysis confirmed the initial tenet of this study, i.e. the necessity of using a high number of strains and differentiated *in vitro* models. Regarding the methodology, the important conclusion can be elucidated from the study, that the more complex applied models (and the more they reflect the actual environment of the chronic wound), the more tolerant biofilms to the used antimicrobials are. In turn, the obstacle of using models of high complexity is that there are higher differences between the lowest and highest values of obtained results because more experimental steps are performed, and the various types of responses biofilms may have in the environment consist of multiple variables.

Despite the wide range of antiseptic agents available for wound treatment, selecting the appropriate antiseptic is still a difficult clinical challenge. There are scientific reports that prove successful therapy with the use of low-concentrated hypochlorites of nasal lavage in persistent rhinosinusitis (Raza et al., 2008), preoperative decolonization by 5-day interventions with intranasal mupirocin and bathing with dilute bleach (a quarter cup of 6% sodium hypochlorite per tub of water) (Fritz et al., 2011), adjuvant use in necrotizing soft tissue infection (Tata et al., 2009), osteitis (Küster et al., 2016) and osteomyelitis (Aragón-Sánchez et al., 2013) or caveats with missing drainage and to reduce the symptomatology and risk of disease progression in ambulatory patients with COVID-19 (Delgado-Enciso et al., 2021). Nevertheless, in the light of data presented in this manuscript, the obtained favorable clinical results of low concentrated hypochlorites should be considered an effect of their rinsing activity combined with low cytotoxicity but not with antimicrobial effect per se. Polyhexanide should be considered the agent of choice for the treatment of heavily biofilm-infected wounds because of its higher efficacy against biofilms and its remanent efficacy.

## Data availability statement

The original contributions presented in the study are included in the article, further inquiries can be directed to the corresponding author/s.

## Author contributions

JP, AJ and MB designed the research. JP, JC, PK, HB, EZ-Z performed all the experiments. JP and AJ and performed the statistical analysis. JP wrote the first draft of the manuscript. JP and AJ analyzed the data and prepared graphics. JC, PK, AK, and HB wrote sections of the manuscript. AJ, MB, AK, and HB, revised and edited the manuscript. AJ and MB supervised the work. All authors contributed to the article and approved the submitted version.

## References

[B1] Alhede M. Kragh K. N. Qvortrup K. Allesen-Holm M. Gennip M. van, Christensen L. D.. (2011). Phenotypes of non-attached pseudomonas aeruginosa aggregates resemble surface attached biofilm. PloS One 6, e27943. doi: 10.1371/journal.pone.0027943 22132176 PMC3221681

[B2] Alves P. J. Barreto R. T. Barrois B. M. Gryson L. G. Meaume S. Monstrey S. J. (2021). Update on the role of antiseptics in the management of chronic wounds with critical colonisation and/or biofilm. Int. Wound J. 18, 342–358. doi: 10.1111/iwj.13537 33314723 PMC8244012

[B3] Aragón-Sánchez J. Lázaro-Martínez J. L. Quintana-Marrero Y. Sanz-Corbalán I. Hernández-Herrero M. J. Cabrera-Galván J. J. (2013). Super-oxidized solution (Dermacyn wound care) as adjuvant treatment in the postoperative management of complicated diabetic foot osteomyelitis: Preliminary experience in a specialized department. Int. J. Low Extrem Wounds 12, 130–137. doi: 10.1177/1534734613476710 23446366

[B4] Barrigah-Benissan K. Ory J. Dunyach-Remy C. Pouget C. Lavigne J.-P. Sotto A. (2022). Antibiofilm properties of antiseptic agents used on pseudomonas aeruginosa isolated from diabetic foot ulcers. Int. J. Mol. Sci. 23, 11270. doi: 10.3390/ijms231911270 36232569 PMC9569737

[B5] Bjarnsholt T. Kirketerp-Møller K. Jensen P.Ø. Madsen K. G. Phipps R. Krogfelt K.. (2008). Why chronic wounds will not heal: A novel hypothesis. Wound Repair Regeneration 16, 2–10. doi: 10.1111/j.1524-475X.2007.00283.x 18211573

[B6] Ciecholewska-Juśko D. Żywicka A. Junka A. Woroszyło M. Wardach M. Chodaczek G.. (2022). The effects of rotating magnetic field and antiseptic on *in vitro* pathogenic biofilm and its milieu. Sci. Rep. 12, 8836. doi: 10.1038/s41598-022-12840-y 35614186 PMC9132948

[B7] Czajkowska J. Junka A. Hoppe J. Toporkiewicz M. Pawlak A. Migdał P.. (2021). The Co-culture of staphylococcal biofilm and fibroblast cell line: The correlation of biological phenomena with metabolic NMR1 footprint. Int. J. Mol. Sci. 22, 5826. doi: 10.3390/ijms22115826 34072418 PMC8198359

[B8] Delgado-Enciso I. Paz-Garcia J. Barajas-Saucedo C. E. Mokay-Ramírez K. A. Meza-Robles C. Lopez-Flores R.. (2021). Safety and efficacy of a COVID-19 treatment with nebulized and/or intravenous neutral electrolyzed saline combined with usual medical care vs. usual medical care alone: A randomized, open-label, controlled trial. Exp. Ther. Med. 22, 915. doi: 10.3892/etm.2021.10347 34306189 PMC8281484

[B9] Eggers M. (2019). Infectious disease management and control with povidone iodine. Infect. Dis. Ther. 8, 581–593. doi: 10.1007/s40121-019-00260-x 31414403 PMC6856232

[B10] Esin S. Kaya E. Maisetta G. Romanelli M. Batoni G. (2022). The antibacterial and antibiofilm activity of granudacyn *in vitro* in a 3D collagen wound infection model. J. Wound Care 31, 908–922. doi: 10.12968/jowc.2022.31.11.908 36367808

[B11] Fritz S. A. Camins B. C. Eisenstein K. A. Fritz J. M. Epplin E. K. Burnham C.-A.. (2011). Effectiveness of measures to eradicate staphylococcus aureus carriage in patients with community-associated skin and soft-tissue infections: A randomized trial. Infect. Control Hosp Epidemiol. 32, 872–880. doi: 10.1086/661285 21828967 PMC3528015

[B12] Gomes L. C. Mergulhão F. J. (2017). SEM analysis of surface impact on biofilm antibiotic treatment. Scanning 8, e2960194. doi: 10.1155/2017/2960194 PMC5662067 29109808

[B13] González J. F. Hahn M. M. Gunn J. S. (2018). Chronic biofilm-based infections: Skewing of the immune response. Pathog. Dis. 76, 1–7. doi: 10.1093/femspd/fty023 PMC6251518 29718176

[B14] Junka A. Bartoszewicz M. Smutnicka D. Secewicz A. Szymczyk P. (2014). Efficacy of antiseptics containing povidone-iodine, octenidine dihydrochloride and ethacridine lactate against biofilm formed by pseudomonas aeruginosa and staphylococcus aureus measured with the novel biofilm-oriented antiseptics test. Int. Wound J. 11, 730–734. doi: 10.1111/iwj.12057 23445335 PMC7950748

[B15] Kampf G. (2016). Acquired resistance to chlorhexidine – is it time to establish an a’ntiseptic stewardship’ initiative? J. Hosp. Infection 94, 213–227. doi: 10.1016/j.jhin.2016.08.018 27671220

[B16] Kragh K. N. Alhede M. Kvich L. Bjarnsholt T. (2019). Into the well–a close look at the complex structures of a microtiter biofilm and the crystal violet assay. Biofilm 1, 1–9. doi: 10.1016/j.bioflm.2019.100006 PMC7798451 33447793

[B17] Kramer A. Dissemond J. Kim S. Willy C. Mayer D. Papke R.. (2018). Consensus on wound antisepsis: Update 2018. Skin Pharmacol. Physiol. 31, 28–58. doi: 10.1159/000481545 29262416

[B18] Krasowski G. Junka A. Paleczny J. Czajkowska J. Makomaska-Szaroszyk E. Chodaczek G.. (2021). *In vitro* evaluation of polihexanide, octenidine and NaClO/HClO-based antiseptics against biofilm formed by wound pathogens. Membranes 11, 62. doi: 10.3390/membranes11010062 33477349 PMC7830887

[B19] Küster I. Kramer A. Bremert T. Langner S. Hosemann W. Beule A. G. (2016). Eradication of MRSA skull base osteitis by combined treatment with antibiotics and sinonasal irrigation with sodium hypochlorite. Eur. Arch. Otorhinolaryngol 273, 1951–1956. doi: 10.1007/s00405-015-3739-x 26227617

[B20] Lappin-Scott H. Burton S. Stoodley P. (2014). Revealing a world of biofilms — the pioneering research of bill costerton. Nat. Rev. Microbiol. 12, 781–787. doi: 10.1038/nrmicro3343 25157698

[B21] Malone M. Bjarnsholt T. McBain A. James G. Stoodley P. Leaper D.. (2017). The prevalence of biofilms in chronic wounds: A systematic review and meta-analysis of published data. J. Wound Care 26, 20–25. doi: 10.12968/jowc.2017.26.1.20 28103163

[B22] Nielsen D. W. Klimavicz J. S. Cavender T. Wannemuehler Y. Barbieri N. L. Nolan L. K.. (2018). The impact of media, phylogenetic classification and e. coli pathotypes on biofilm formation in extraintestinal and commensal e. coli from humans and animals. Front. Microbiol. 9. doi: 10.3389/fmicb.2018.00902 PMC5951942 29867813

[B23] Paleczny J. Dudek-Wicher R. Dydak K. Oleksy-Wawrzyniak M. Madziała M. Bartoszewicz M.. (2022). The medium composition impacts staphylococcus aureus biofilm formation and susceptibility to antibiotics applied in the treatment of bone infections. Int. J. Mol. Sci. 23, 11564, 1–21. doi: 10.3390/ijms231911564 36232864 PMC9569719

[B24] Paleczny J. Junka A. Brożyna M. Dydak K. Oleksy-Wawrzyniak M. Ciecholewska-Juśko D.. (2021). The high impact of staphylococcus aureus biofilm culture medium on *In vitro* outcomes of antimicrobial activity of wound antiseptics and antibiotic. Pathogens 10, 1385, 1–26. doi: 10.3390/pathogens10111385 34832540 PMC8626063

[B25] Pham L. H. P. Ly K. L. Colon-Ascanio M. Ou J. Wang H. Lee S. W.. (2023). Dissolvable alginate hydrogel-based biofilm microreactors for antibiotic susceptibility assays. Biofilm 5, 100103. doi: 10.1016/j.bioflm.2022.100103 36691521 PMC9860113

[B26] Probst S. Apelqvist J. Bjarnsholt T. Lipsky B. A. Ousey K. Peters E. J. G. (2022). Antimicrobials and Non-Healing Wounds: An Update. (Journal of Wound Management; Vol. 23, No. S1, p. 36). European Wound Management Association (EWMA). https://www.amr-insights.eu/antimicrobials-and-non-healing-wounds-an-update/

[B27] Raza T. Elsherif H. S. Zulianello L. Plouin-Gaudon I. Landis B. N. Lacroix J. S. (2008). Nasal lavage with sodium hypochlorite solution in staphylococcus aureus persistent rhinosinusitis. Rhinology 46, 15–22. 18444487

[B28] Rembe J.-D. Huelsboemer L. Plattfaut I. Besser M. Stuermer E. K. (2020). Antimicrobial hypochlorous wound irrigation solutions demonstrate lower anti-biofilm efficacy against bacterial biofilm in a complex in-vitro human plasma biofilm model (hpBIOM) than common wound antimicrobials. Front. Microbiol. 11. doi: 10.3389/fmicb.2020.564513 PMC7583357 33162949

[B29] Sen C. K. (2019). Human wounds and its burden: An updated compendium of estimates. Adv. Wound Care 8, 39–48. doi: 10.1089/wound.2019.0946 PMC6389759 30809421

[B30] Severing A.-L. Borkovic M. Stuermer E. K. Rembe J.-D. (2022). Composition of challenge substance in standardized antimicrobial efficacy testing of wound antimicrobials is essential to correctly simulate efficacy in the human wound micro-environment. Biomedicines 10, 2751. doi: 10.3390/biomedicines10112751 36359272 PMC9687328

[B31] Severing A.-L. Rembe J.-D. Koester V. Stuermer E. K. (2019). Safety and efficacy profiles of different commercial sodium hypochlorite/hypochlorous acid solutions (NaClO/HClO): Antimicrobial efficacy, cytotoxic impact and physicochemical parameters *in vitro* . J. Antimicrobial Chemotherapy 74, 365–372. doi: 10.1093/jac/dky432 30388236

[B32] Stuermer E. K. Besser M. Brill F. Geffken M. Plattfaut I. Severing A. L.. (2021). Comparative analysis of biofilm models to determine the efficacy of antimicrobials. Int. J. Hygiene Environ. Health 234, 113744. doi: 10.1016/j.ijheh.2021.113744 33780904

[B33] Tata M. D. Kwan K. C. Abdul-Razak M. R. Paramalingam S. Yeen W. C. (2009). Adjunctive use of superoxidized solution in chest wall necrotizing soft tissue infection. Ann. Thorac. Surg. 87, 1613–1614. doi: 10.1016/j.athoracsur.2008.10.019 19379926

[B34] Thaarup I. C. Iversen A. K. S. Lichtenberg M. Bjarnsholt T. Jakobsen T. H. (2022). Biofilm survival strategies in chronic wounds. Microorganisms 10, 775. doi: 10.3390/microorganisms10040775 35456825 PMC9025119

[B35] To E. Dyck R. Gerber S. Kadavil S. Woo K. Y. (2016). The effectiveness of topical polyhexamethylene biguanide (PHMB) agents for the treatment of chronic wounds: A systematic review. Surg. Technol. Int. 29, 45–51. 27608742

[B36] Versey Z. da Cruz Nizer W. S. Russell E. Zigic S. DeZeeuw K. G. Marek J. E.. (2021). Biofilm-innate immune interface: Contribution to chronic wound formation. Front. Immunol. 12. doi: 10.3389/fimmu.2021.648554 PMC8062706 33897696

[B37] Vyas H. K. N. Xia B. Mai-Prochnow A. (2022). Clinically relevant *in vitro* biofilm models: A need to mimic and recapitulate the host environment. Biofilm 4, 100069. doi: 10.1016/j.bioflm.2022.100069 36569981 PMC9782257

[B38] Woroszyło M. Ciecholewska-Juśko D. Junka A. Pruss A. Kwiatkowski P. Wardach M.. (2021). The impact of intraspecies variability on growth rate and cellular metabolic activity of bacteria exposed to rotating magnetic field. Pathogens 10, 1427. doi: 10.3390/pathogens10111427 34832583 PMC8624435

[B39] Yang J. Cheng S. Li C. Sun Y. Huang H. (2019). Shear stress affects biofilm structure and consequently current generation of bioanode in microbial electrochemical systems (MESs). Front. Microbiol. 10. doi: 10.3389/fmicb.2019.00398 PMC6415583 30894842

